# Workplace aggression against healthcare workers in a Spanish healthcare institution between 2019 and 2021: The impact of the COVID-19 pandemic

**DOI:** 10.3389/fpubh.2023.1070171

**Published:** 2023-03-22

**Authors:** Aitor Díaz, Mireia Utzet, Joan Mirabent, Pilar Diaz, Jose Maria Ramada, Consol Serra, Fernando G. Benavides

**Affiliations:** ^1^Center for Research in Occupational Health, Department of Medicine and Life Sciences, Universitat Pompeu Fabra, Barcelona, Spain; ^2^IMIM-Hospital del Mar Medical Research Institute, Barcelona, Spain; ^3^CIBER of Epidemiology and Public Health, Madrid, Spain; ^4^Occupational Health Service, Parc de Salut Mar, Barcelona, Spain

**Keywords:** healthcare workers (HCW), COVID-19 pandemic, cohort, Spain, workplace aggression

## Abstract

**Objectives:**

Describe the incidence of first aggressions among healthcare workers (HCWs) before and during the COVID-19 pandemic in a Spanish healthcare institution, according to workers' socio-occupational characteristics and analyze the impact of the pandemic on it.

**Methods:**

A cohort involving HCWs who worked in the institution for at least 1 week each year from 1 January 2019 to 31 December 2021. Adjusted relative risks (aRR) were estimated using generalized estimating equations and negative binomial models to calculate the differences in WPA between the different time periods. All analyses were stratified by gender.

**Results:**

Among women, the incidence was 6.8% (6.0; 7.8) during the pre-COVID-19 period, 6.0% (5.2; 7.0) during the COVID-19 baseline and 5.1% (4.3; 5.9) during the COVID-19 endline; and 4.6% (3.4; 6.1), 5.3% (4.1; 6.8) and 4.4% (3.5; 5.8), respectively, among men. Among men, the incidence of WPA was 4.6 (3.4; 6.1), 5.3 (4.1; 6.8), and 4.4% (3.5; 5.8), respectively. These incidences were significantly higher among male nurses and aides [11.1 (8.0; 15.4), 12.3 (8.9; 16.6), and 9.3% (6.5; 13.3) during each period] and psychiatric center workers [women: 14.7 (11.2; 19.0), 15.4 (11.8; 19.8), and 12.4% (9.2; 16.6); men: 12.3 (7.2; 20.0), 17.8 (11.6; 26.2), and 14.3% (8.8; 22.4)]. Among women, the risk of WPA was 23% lower in the post-COVID-19 period compared to before the pandemic [aRR = 0.77 (0.64; 0.93)], while the risk during the COVID-19 baseline was not significantly different [aRR = 0.89 (0.74; 1.06)].

**Conclusions:**

The COVID-19 pandemic led to an unexpected decrease in first-time WPA against HCWs. However, ~5% of HCWs experienced at least one incidence of aggression in the last follow-up year. Healthcare managers should continue to increase the prevention of aggression against HCWs, especially among vulnerable groups with a higher level of incidence.

## Introduction

Workplace aggression (WPA) against healthcare workers (HCWs) is a well-known occupational health challenge that threatens the wellbeing of workers and their right to work in a safe environment. Although violence is a worldwide phenomenon that may occur in any workplace, several studies have shown that HCWs are at a greater risk of experiencing violence at work (1). As per the guidelines of the World Health Organization and International Labor Organization, in this study, we defined WPA (2, 3) as an intentional act that is either verbal and/or physical (4) and is committed by patients or their family members, which can cause both physical injury and psychological harm.

During the last few decades, scientific interest in WPA against HCWs has grown substantially (5). A systematic review published at the end of 2019 reported a high frequency of workplace violence by patients and visitors against nurses and physicians, with more than half of the HCWs reporting that they experienced violence at some point over the course of their careers (6). Workers in psychiatric and emergency department settings, and those working long hours, and nursing staff have been shown to be more vulnerable to WPA (7–9). However, WPA is often underreported in healthcare, partly due to the normalization of violence. Consequently, its real magnitude is underestimated, which can lead to insufficient or inappropriate efforts to address this issue (10).

WPA in the healthcare sector can be triggered by several determinants, such as sociodemographic and health variables (both in the aggressor and the victim) and factors related to the workplace and working conditions (7). However, the causes and consequences of WPA are not always clear (11). Within the multi-causal etiology of WPA in the health sector, the COVID-19 pandemic may have had an impact on its incidence. During the first few months of the pandemic, HCWs were applauded daily (in Spain and many other countries) and thanked for their help and dedication to patients (12). However, there is evidence that the COVID-19 pandemic, similar to other health emergencies, has led to an increase in WPA toward HCWs (13, 14). It has been shown that a lack of technical and human resources, deterioration of the healthcare system, and mistrust surrounding COVID-19 have led to prejudices toward HCWs and the tasks they perform. This, in turn, could have contributed to the emergence of new types of WPA in the context of a health emergency (15). After analyzing cross-sectional data from the first months of the pandemic through an online and global survey in four languages, Dye et al. (16) found that the professionals working in healthcare settings were significantly more likely to experience COVID-19-related harassment, bullying, or harm. Other studies suggest that WPA against HCWs was highly prevalent during the COVID-19 pandemic (17). Furthermore, several factors that may increase the prevalence of WPA during the pandemic have been identified, including being a nurse aide or a technician, being infected with COVID-19, or caring for patients infected with COVID-19 (18).

In this context, and in line with previous literature, our initial hypothesis was that the pandemic context could have increased WPA against HCWs. However, to the best of our knowledge, there are almost no studies analyzing the time trend of the prevalence of WPA against HCWs and the role of the COVID-19 pandemic in this phenomenon. The objectives of this study were as follows: 1) to describe the incidence of first WPA among HCWs before (2019) and during (2020 and 2021) the COVID-19 pandemic in a Spanish healthcare institution according to the workers' socio-demographic and occupational characteristics; and 2) to analyze the possible impact of the pandemic on the incidence of WPA against HCWs by their occupational characteristics.

## Materials and methods

### Study design and population size

This study was conducted using a retrospective cohort from 1 January 2019 to 31 December 2021 at the Parc de Salut Mar (PSMar), a healthcare institution located in Barcelona, Spain. This institution has 1,902 beds, 33,000 annual discharges, and eight health centers. This study adopted a longitudinal panel design, and only staff who worked for at least 1 week each year (2019, 2020, and 2021) were included in the analysis. Therefore, the sample analyzed remained constant throughout the study period (see Supplementary Figure 1, Supplementary Table S1).

Information on HCW was available from the Human Resources Department databases. For each HCW, we retrieved sociodemographic and occupational information. In addition, the occupational health service provided us with a database with all reports of aggression against healthcare workers. The data were obtained from a self-reported questionnaire completed by a worker who had experienced aggression. A participant identification number for the study was created to link both databases and ensure confidentiality. Privacy and data safety were guaranteed, and the PSMar Ethics Committee approved the study (20 July 2022, number 2022_10465).

### Inclusion and exclusion criteria

The inclusion criteria were as follows: (1) subject being an HCW, (2) subjects engaged in direct patient care or not, subjects aged 18–70 years, and subjects having been employed for at least 1 week each year during the study period. The exclusion criteria were healthcare staff with a contractual relationship with the hospital of <1 week per year during the study period or staff working in the hospital but contracted by an external company.

### Definitions and information on variables

The main response variable was whether or not the HWC had experienced the first incidence of WPA by patients or their family members each year. If an HCW suffered more than one incidence of WPA per year, we only included the first case (see Supplementary Figure 1). Each incidence of aggression was classified by the type of WPA (verbal or physical). The incidents registered as both verbal and physical were reclassified as physical.

The time period was categorized as “the pre-COVID-19 period” (2019), “COVID-19 baseline” (2020), and “the post-COVID-19 period” (2021).

For each worker, we included information on the type of contract (permanent, temporary, or replacement); occupational category (physicians, nurses, and nurse aides, other HCW, such as medical and other trainees or lab technicians and administration and management staff); work shift (day or night); and center within PSMar, which are Hospital del Mar (acute care), Hospital de l'Esperança (acute care), Center Fòrum (long-term care and psychiatry), and the Dr. Emili Mira Center (psychiatry). Sociodemographic variables were also included: sex and age (18–29, 30–49, and 50–70 years).

### Statistical analysis

The study variables for each period were described as sample counts and percentages stratified by sex, and their significance was tested using the chi-squared test. The cumulative incidence (I) per 100 first cases of WPA and their 95% confidence intervals (95% CI) were estimated according to sociodemographic and occupational variables and stratified by sex and time period. The homogeneity chi-square test was used to assess possible differences in the incidence of WPA among the categories of each variable of interest. The crude relative risks (cRR) and their 95% confidence intervals (95% CI) between time periods (COVID-19 baseline and post-COVID-19 vs. pre-COVID-19 period) were calculated according to sociodemographic and occupational variables and stratified by sex. Second, the adjusted relative risks (aRR) and 95% CI between the three time periods (taking the pre-COVID19 year as reference) were estimated with a generalized estimating equations negative binomial model with log link and the “unstructured” working correlation matrix, as it provided the best fit for the data (19). Five consecutive approaches were used: (1) fitting a crude baseline by model introducing a dummy variable into the model identifying the time period (pre-pandemic and during the pandemic), taking the first approach as a reference, (2) including the age in the crude model the age, (3) including the occupational category in the crude model, and (4) including the type of contract, work shift, and center within PSMar in the crude model. These four approaches were estimated by stratifying by sex. All calculations were conducted with STATA version 14.2 (Stata Corp., College Station, TX, USA).

## Results

The final sample consisted of 3,884 individuals; three-quarters of whom were women (74.1%). During the three periods and for both women and men, the age distribution changed significantly, but half of the staff (around 47%) was middle-aged (see Table 1). In all three periods, most of the individuals in the sample (80%) had a permanent contract, worked the day shift (around 84%), and worked at the Hospital del Mar (about 70%); around half of the women were nursing staff, with the rest of the professional categories being similar (~15%), while men were equally distributed among the four professional categories (see Table 2). Only the contract arrangements of HCWs changed significantly during the three periods, with those on temporary contracts decreasing significantly during the pandemic.

**Table 1:** Healthcare workers sociodemographic (*n*, %) by time period^*^ and sex.

**Total (*****n*** = **3,884)**				
		**Pre-COVID-19**	**COVID-19 baseline**	**COVID-19 endline**
Age (years)	18–29	924 (23.8)	829 (21.3)	722 (18.6)
	30–50	1,832 (47.2)	1,854 (47.7)	1,873 (48.2)
	51–70	1,128 (29.0)	1,201 (30.9)	1,289 (33.2)
	*p*-value	0		
**Women (*****n*** = **2,879)**				
		**Pre-COVID-19**	**COVID-19 baseline**	**COVID-19 endline**
Age (years)	18–29	659 (22.9)	598 (20.8)	520 (18.1)
	30–50	1,365 (47.4)	1,377 (47.8)	1,394 (48.4)
	51–70	855 (29.7)	904 (31.4)	965 (33.5)
	*p*-value	0,011		
**Men (*****n*** = **1,005)**				
		**Pre-COVID-19**	**COVID-19 baseline**	**COVID-19 endline**
Age (years)	18–29	265 (26.4)	231 (23.0)	202 (20.1)
	30–50	467 (46.5)	477 (47.5)	479 (47.7)
	51–70	273 (27.2)	297 (29.6)	324 (32.2)
	*p*-value	0		

* Pre-COVID-19, COVID-19 baseline, post-COVID-19 periods.

**Table 2:** Healthcare workers occupational variables (*n*, %) by time period^*^ and sex.

		**Women (*****n*** = **2,879)**			**Men (*****n*** = **1,005)**		
		**Pre-COVID-19**	**COVID-19 baseline**	**COVID-19 endline**	**Pre-COVID-19**	**COVID-19 baseline**	**COVID-19 endline**
Type of contract	Permanent	2,233 (77.9)	2,287 (79.4)	2,323 (80.7)	775 (77.3)	804 (80.0)	811 (80.7)
	Temporary	326 (7.5)	80 (2.8)	80 (2.8)	78 (7.8)	33 (3.3)	30 (3.0)
	Replacement	422 (14.7)	512 (17.8)	476 (16.5)	150 (15.0)	168 (16.7)	164 (16.3)
	*p*-value^(a)^	0			0		
Occupational category	Physicians	366 (12.7)	378 (13.1)	401 (13.9)	277 (27.6)	281 (28.0)	291 (29.0)
	Nurses and aides	1,784 (62.0)	1,787 (62.1)	1,788 (62.1)	287 (28.6)	285 (28.4)	289 (28.8)
	Other healthcare workers	343 (11.9)	329 (11.4)	305 (10.6)	264 (26.3)	263 (26.2)	250 (24.9)
	Administration and management staff	386 (13.4)	385 (4.5)	385 (13.4)	177 (17.6)	176 (17.5)	175 (17.4)
	*p*-value^(a)^	0.686			0.99		
Work shift	Day	2,382 (83.5)	2,380 (83.5)	2,275 (82.4)	858 (86.6)	856 (86.7)	838 (86.5)
	Night	471 (16.5)	469 (16.5)	486 (17.6)	133 (13.4)	131 (13.3)	131 (13.5)
	*p*-value^(a)^	0.436			0.987		
Health center	Hospital del Mar (acute care)	1,877 (69.3)	1,869 (69.0)	1,868 (69.2)	699 (73.9)	708 (74.6)	713 (75.4)
	Hospital Esperança (acute care)	273 (10.1)	279 (10.3)	279 (10.3)	81 (8.6)	80 (8.4)	79 (8.4)
	Fòrum center (long-term care, psychiatric care)	238 (8.8)	240 (8.9)	237 (8.8)	60 (6.3)	54 (5.7)	48 (5.1)
	Dr. Emili Mira Center (psychiatric care)	320 (11.8)	319 (11.8)	314 (11.6)	106 (11.2)	107 (11.3)	105 (11.1)
	*p*-value^(a)^	1			0.989		

* Pre-COVID-19, COVID-19 baseline, post-COVID-19 periods.

(a) p-value of the chi-square test.

Throughout the study period, 660 first episodes of WPA were registered, with an incidence rate (and 95% CI) of aggression of 6.8% (6.0; 7.8) during the pre-COVID-19 period, 6.0% (5.2; 7.0) during the COVID-19 baseline, and 5.1% (4.3; 5.9), and the post-COVID-19 period among women. For men, the incidence rates were 4.6 (3.4; 6.1), 5.3 (4.1; 6.8), and 4.4% (3.5; 5.8), respectively (see Table 3). Among women and men, verbal WPA was significantly higher than physical WPA in all three periods (see Supplementary Table S2). When stratified by sociodemographic and occupational variables (see Table 3), the incidence of new cases of WPA was found to be significantly higher among male and female nurses and aides (although among the latter, the difference was not statistically significant) compared to other occupational categories. The incidence of WPA was also significantly higher among both female and male workers in the psychiatric center (the Dr. Emili Mira Center) than among workers in other centers during all three periods.

**Table 3:** Incident episodes (*n*, %), cumulative incidence (*I*) and 95% confidence intervals (95% CI) of aggression according to sociodemographic and occupational variables, by time period^*^ and sex.

		**Women**						**Men**					
		**Pre-COVID-19**		**COVID-19 baseline**		**COVID-19 endline**		**Pre-pandemic**		**Pre-COVID-19**		**COVID-19 baseline**	
		***n*** **(%)**	***I*** **(95% CI)**	***n*** **(%)**	***I*** **(95% CI)**	***n*** **(%)**	***I*** **(95% CI)**	***n*** **(%)**	***I*** **(95% CI)**	***n*** **(%)**	***I*** **(95% CI)**	***n*** **(%)**	***I*** **(95% CI)**
Total		197	6.8	174	6.0	146	5.1	46	4.6	53	5.3	44	4.4
			(6.0; 7.8)		(5.2; 7.0)		(4.3; 5.9)		(3.4; 6.1)		(4.1; 6.8)		(3.3; 5.8)
Age (years)	18–30	50 (25.4)	7.6	40 (23.0)	6.7	42 (28.8)	8.1	14 (30.4)	5.3	15 (28.3)	6.5	10 (22.7)	5.0
				(5.8; 9.9)		(4.9; 9.0)		(6.0; 10.8)		(3.2; 8.7)		(3.9; 10.5)		(2.7; 9.0)
	31–50	97 (49.2)	7.1	94 (54.0)	6.8	67 (45.9)	4.8	27 (58.7)	5.8	29 (54.7)	6.1	27 (61.4)	5.6
				(5.9; 8.6)		(5.6; 8.3)		(3.8; 6.1)		(4.0; 8.3)		(4.3; 8.6)		(3.9; 8.1)
51–70	50 (25.4)	5.8	40 (23.0)	4.4	37 (25.3)	3.8	5 (10.9)	1.8	9 (17.0)	3.0	7 (15.9)	2.2
			(4.5; 7.6)		(3.3; 6.0)		(2.9; 5.2)		(0.8; 4.3)		(1.6; 5.7)		(1.0; 4.5)
*p*-value^(a)^	0.359		0.047		0.001		0.038		0.116		0.056	
Type of contract	Permanent	149 (75.6)	6.7	127 (73.0)	5.6	111 (76.0)	4.8	31 (67.4)	4.0	37 (69.8)	4.6	33 (75.0)	4.1
				(5.7; 7.8)		(4.7; 6.6)		(4.0; 5.7)		(2.8; 5.6)		(3.4; 6.3)		(2.9; 5.7)
	Temporary	18 (9.1)	8.4	3 (1.7)	3.8	1	1.3	3 (6.5)	3.8	2 (3.8)	6.1	0	-
				(5.3; 12.9)		(1.2; 11.1)	(0.7)	(0.2; 8.4)		(1.2; 11.3)		(1.5; 21.6)		
Replacement	29 (14.7)	6.9	44 (25.3)	8.6	34 (23.3)	7.1	12 (26.1)	8.0	14 (26.4)	8.3	11 (25.0)	6.7
			(4.8; 9.7)		(6.5; 11.4)		(5.1; 9.8)		(4.6; 13.6)		(5.0; 13.6)		(3.7; 11.7)
*p*-value^(a)^	0.64		0.023		0.029			0.095	0.141		0.159	
Occupational category	Physicians	11 (5.6)	3.0	17 (9.8)	4.5	10 (6.8)	2.5	3 (6.5)	1.1	3 (5.7)	1.1	2 (4.5)	0.7
				(1.7; 5.3)		(2.8; 7.1)		(1.3; 4.6)		(0.3; 3.3)		(0.3; 3.3)		(0.2; 2.7)
	Nurses and aides	161 (81.7)	9.0	136 (78.2)	7.6	111 (76.0)	6.2	32 (69.6)	11.1	35 (66.0)	12.3	27 (61.4)	9.3
				(7.8; 10.4)		(6.5; 8.9)		(5.2; 7.4)		(8.0; 15.4)		(8.9; 16.6)		(6.5; 13.3)
	Other healthcare workers	12 (6.1)	3.5	17 (9.8)	5.2	14 (9.6)	4.6	6 (13.0)	2.3	9 (17.0)	3.4	9 (20.5)	0.4
			(2.0; 6.1)		(3.2; 8.2)		(2.7; 7.6)		(1.0; 5.0)		(1.8; 6.5)		(1.9; 6.8)
Administration and management staff	13 (6.6)	3.4	4 (2.3)	1.0	11 (7.5)	2.9	5 (10.9)	2.8	6 (11.3)	3.4	6 (13.6)	3.4
			(2.0; 5.7)		(0.4; 2.7)		(1.6; 5.1)		(1.2; 6.6)		(1.5; 7.4)		(0.2; 7.4)
*p*-value^(a)^	0.000		0.000		0.002		0.000		0.000		0.000	
Work shift	Day	162 (82.2)	6.8	140 (80.5)	5.9	115 (78.8)	6.2	34 (73.9)	4.0	39 (73.6)	4.6	36 (81.8)	4.3
				(5.9; 7.9)		(5.0; 6.9)		(4.3; 8.7)		(2.8; 5.5)		(3.3; 6.2)		(3.1; 5.9)
	Night	34 (17.3)	7.2	33 (19.0)	7.0	30 (20.5)	0.8	11 (23.9)	8.3	12 (22.6)	9.2	7 (15.9)	5.3
			(5.2; 9.9)		(5.0; 9.7)		(0.1; 5.8)		(4.6; 14.3)		(5.3; 15.5)		(2.6; 10.8)
*p*-value^(a)^	0.788		0.339		0.061		0.078		0.027		0.769	
Health center	Hospital del Mar (acute care)	104 (52.8)	5.5	84 (48.3)	4.5	76 (52.1)	4.1	24 (52.2)	3.4	23 (43.4)	3.2	21 (47.7)	2.9
				(4.6; 6.7)		(3.6; 5.5)		(3.3; 5.1)		(2.3; 5.1)		(2.2; 4.8)		(1.9; 4.5)
	Hospital Esperança (acute care)	12 (6.1)	4.4	8 (4.6)	2.9	6 (4.1)	2.2	0	-	0	-	1 (2.3)	1.3
				(2.5; 7.6)		(1.4; 5.6)		(1.0; 4.7)						(0.2; 8.5)
	Fòrum center (long-term care and, psychiatric care)	18 (9.1)	7.6	18 (10.3)	7.5	16 (11.0)	6.8	2 (4.3)	3.3	4 (7.5)	7.4	3 (6.8)	6.2
			(4.8; 11.7)		(4.8; 11.6)		(4.2; 10.7)		(0.8; 12.5)		(2.8; 18.3)		(2.0; 17.8)
Dr. Emili Mira Center (psychiatric care)	47 (23.9)	14.7	49 (28.2)	15.4	39 (26.7)	12.4	13 (28.3)	12.3	19 (35.8)	17.8	15 (34.1)	14.3
			(11.2; 19.0)		(11.8; 19.8)		(9.2; 16.6)		(7.2; 20.0)		(11.6; 26.2)		(8.8; 22.4)
*p*-value^(a)^	0.000		0.000		0.000		0.000		0.000		0.000	

* Pre-COVID-19, COVID-19 baseline, post-COVID-19 periods.

(a) p-value of the chi-square test for homogeneity.

After stratifying the study variables by time period (see Table 4), significant changes were found only among women while comparing the post-COVID-19 period with the pre-COVID-19 period. There was a significant 24% decrease [cRR = 0.76 (0.61; 0.93)] in the incidence of new WPA among HCW during the post-COVID-19 period, particularly in the case of verbal WPA [0.70 (0.54; 0.90)], among workers aged 31–50 [0.67 (0.50; 0.92)] and 51–70 years [0.66 (0.43; 0.99)], those with a permanent contract [0.72 (0.56; 0.91)], nurses and aides [0.69 (0.55; 0.87)], and workers in the acute care center Hospital del Mar [0.73 (0.55; 0.98)]. Comparing the COVID-19 baseline with the pre-COVID-19 period revealed a significant decrease in WPA among administrative and management staff [0.31 (0.10; 0.94)].

**Table 4:** Crude relative risk (cRR) and 95% confidence intervals (95% CI) of aggressions between time periods^*^ according to type, sociodemographic and occupational variables, by sex.

		**Women**		**Men**	
		**COVID-19 baseline vs. pre-COVID-19**	**COVID-19 endline vs. pre-COVID-19**	**COVID-19 baseline vs. pre-COVID-19**	**COVID-19 endline vs. pre-COVID-19**
		**cRR (95% CI)**	**cRR (95% CI)**	**cRR (95% CI)**	**cRR (95% CI)**
Total		0.90 (0.74; 1.10)	0.76 (0.61; 0.93)	1.15 (0.78; 1.69)	0.96 (0.64; 1.43)
Type of aggression	Verbal	0.83 (0.65; 1.06)	0.70 (0.54; 0.90)	1.2 (0.71; 2.03)	1.24 (0.74; 2.08)
	Physical	1−	0.84 (0.58; 1.21)	1.10 (0.61; 1.97)	0.62 (0.31; 1.23)
Age (years)	18–30	0.88 (0.59; 1.32)	1.06 (0.72; 1.58)	1.23 (0.61; 2.49)	0.94 (0.43; 2.07)
	31–50	0.96 (0.73; 1.26)	0.67 (0.50; 0.92)	1.05 (0.63; 1.75)	0.97 (0.58; 1.64)
	51–70	0.76 (0.50; 1.13)	0.66 (0.43; 0.99)	1.65 (0.56; 4.88)	1.18 (0.38; 3.67)
Type of contract	Permanent	0.83 (0.66; 1.05)	0.72 (0.56; 0.91)	1.15 (0.72; 1.84)	1.02 (0.63; 1.64)
	Temporary	0.68 (0.21; 2.25)	0.23 (0.03; 1.67)	1.58 (0.28; 9.00)	0
	Replacement	1.25 (0.80; 1.96)	1.04 (0.64; 1.68)	1.04 (0.50; 2.18)	0.84 (0.38; 1.84)
Occupational category	Physicians	1.50 (0.71; 3.15)	0.83 (0.36; 1.93)	0.99 (0.20; 4.84)	0.63 (0.11; 3.77)
	Nurses and aides	0.84 (0.68; 1.05)	0.69 (0.55; 0.87)	1.10 (0.70; 1.73)	0.84 (0.52; 1.36)
	Other health care workers	1.48 (0.72; 3.04)	1.31 (0.61; 2.79)	1.51 (0.54; 4.17)	1.58 (0.57; 4.39)
	Administration and management staff	0.31 (0.10; 0.94)	0.85 (0.38; 1.87)	1.21 (0.38; 3.88)	1.21 (0.38; 3.90)
Work shift	Day	0.92 (0.81; 1.05)	0.74 (0.59; 0.94)	1.15 (0.73; 1.80)	1.08 (0.69; 1.72)
	Night	0.97 (0.61; 1.55)	0.86 (0.53; 1.37)	1.11 (0.51; 2.42)	0.65 (0.26; 1.62)
Health center	Hospital del Mar (acute care)	0.81 (0.61; 1.07)	0.73 (0.55; 0.98)	0.95 (0.54; 1.66)	0.86 (0.48; 1.53)
	Hospital Esperança (acute care)	0.65 (0.27; 1.57)	0.49 (0.19; 1.29)	–	–
	Fòrum center (long-term care, psychiatric care)	0.99 (0.53; 1.86)	0.89 (0.47; 1.71)	2.22 (0.42; 11.65)	1.88 (0.33; 10.77)
	Dr. Emili Mira Center (psychiatric care)	1.05 (0.72; 1.51)	0.85 (0.57; 1.26)	1.45 (0.75; 2.78)	1.16 (0.58; 2.33)

* COVID-19 baseline and COVID-19 endline vs. pre-COVID-19.

Among the women, the adjusted risk (Table 5) for the first-time WPA during the post-COVID-19 period was 23% lower than during the pre-COVID-19 period [RR = 0.77 (0.64; 0.93)], with almost no difference between the crude and fully adjusted models. The risk during the COVID-19 baseline did not differ from that before the pandemic [RR = 0.89 (0.74; 1.06)]. Among the men, there was no significant difference between the time periods.

**Table 5:** Relative risk (RR) and 95% confidence interval (95% CI) of aggressions between time periods^*^, according to sociodemographic and occupational variables, by sex.

		**Model 1**	**Model 2**	**Model 3**	**Model 4**	**Model 5**
		**RR (95% CI)**	**RR (95% CI)**	**RR (95% CI)**	**RR (95% CI)**	**RR (95% CI)**
Women	Pre-COVID-19	1	1	1	1	1
	COVID-19 baseline	0.88 (0.74; 1.05)	0.89 (0.75; 1.06)	0.88 (0.74; 1.05)	0.88 (0.74; 1.05)	0.89 (0.74; 1.06)
	COVID-19 endline	0.74 (0.62; 0.89)	0.75 (0.62; 0.91)	0.74 (0.62; 0.90)	0.76 (0.63; 0.91)	0.77 (0.64; 0.93)
Men	Pre-COVID-19	1	1	1	1	1
	COVID-19 baseline	1.15 (0.84; 1.58)	1.18 (0.86; 1.62)	1.18 (0.86; 1.62)	1.11(0.80; 1.55)	1.13 (0.81; 0.548)
	COVID-19 endline	0.96 (0.69; 1.33)	0.99 (0.71; 1.39)	0.97 (0.69; 1.37)	0.96 (0.68; 1.36)	0.97 (0.68; 1.39)

* COVID-19 baseline and poast-COVID-19 vs. pre-COVID-19 periods.

Model 1: crude model. Model 2: Model 1, including age. Model 3: Model 1, including occupational category. Model 4: Model 1, including type of contract, work shift and health center; Model 5: Model 1, including age, occupational category, type of contract, work shift and center.

## Discussion

This study's main and unexpected result was that the risk of first-time WPA posed by patients or family members decreased among the HCWs of PSMar during the COVID-19 pandemic compared with the pre-COVID-19 period. However, this statistically significant decrease occurred only during the post-COVID-19 period and among women (who represented 75% of the sample), workers older than 30 years, nursing staff, those working the day shift, and those with a permanent contract. Furthermore, we identified several groups that were vulnerable to aggression, namely, those younger than 50 years, those with a replacement contract, nursing and aide staff, and staff in the psychiatric center.

The main result was the opposite of our initial hypothesis that the risk of the first-time WPA increased during the pandemic, as reported by most recent studies (13, 15). A possible explanation for this difference is that, in contrast to the present data, none of those studies compared data on the incidence of WPA before and during the pandemic. However, a recent study analyzing data from 2020 and 2021 also found a decrease in the prevalence of WPA among HCWs the year after the onset of the pandemic (20).

Our main finding has several possible explanations. First, during the pandemic years, health workers' workload and emotional exhaustion eased dramatically (21). This could have led to a decrease in the reporting of WPA because of the time-consuming reporting procedures involved (10). Second, this reduction could be explained by the possible decrease in hospital visits due to the restrictions of the pandemic. However, data on all hospital attendance (admissions, interventions, visits to the emergency department, tests performed, and so on [data not shown]) demonstrate that the trend, while decreasing in 2020, increased in 2021, when hospital attendance was even higher than that in 2019.

Furthermore, during the pandemic, positive collective attitudes toward HCW were enhanced by the emphasis placed, especially by the media, on the daily efforts of HCW during the most virulent moments of the pandemic, similar to what occurred in most countries (22). This positive attitude may have encouraged certain social skills among patients (such as respect or assertiveness) to appear more frequently in this type of context. Finally, the results may be partially explained by the adoption of organizational prevention measures during the pandemic. Outpatient activity (outpatient consultations, complementary examinations, and day hospitals) was drastically reduced, and only professionals made vulnerable because of their health conditions and those older than 50 years were retained in these areas. This drastically reduced waiting times for patients in areas with a high risk of WPA, which, together with the majority presence of experienced professionals, could be the reason for the decrease in WPA during the pandemic period. All these alternative hypotheses should be confirmed by further studies. Other specific results could also help to understand and prevent this occupational risk factor. Our results showed that ~6.8% (6.0; 7.8) of women reported experiencing at least one incidence of WPA during the pre-pandemic year, which decreased to 6.0% (5.2; 7.0) during the first year of the pandemic and to 5.1% (4.3; 5.9) during the second year. Among the men, the pattern differed, starting at 4.6% (3.4; 6.1) during the pre-pandemic period, increasing to 5.3% (4.1; 6.8) during the first year of the pandemic, and decreasing to 4.4% (3.5; 5.8) during the second year. These results are similar to the reported incidence (5.7%) of a cross-sectional study among Italian HCWs during the first year of the pandemic (22) but are significantly lower than those reported by other studies. For instance, a study in Spain showed that 10% of nurses experienced WPA (23); a study based on an Italian HCW cohort found a 12-month incidence of new WPA of around 24% (24); and a meta-analysis conducted in 2019 (8) found a 12-month prevalence of WPA among HCW of 48.1% in Europe. These differences may be partly explained by the considerable variability in the definitions of aggression, the procedure for recording aggression, and the questionnaires used in the various literature studies, which all hamper comparisons.

Furthermore, in this study, to accurately quantify the incidence rate, we did not include repeat aggression by a single professional during the study period. Finally, WPA may have been underreported. As suggested in the literature, there are various reasons for the underreporting of WPA, such as time-consuming incident reporting procedures, inadequate supervisory or coworker support, or beliefs that reporting cases of violence will not lead to any positive changes (8). However, in our hospital, a simple self-recording system is available to professionals to report WPA.

Our results on the incidence of the first WPA among HCWs in terms of demographic and occupational variables show that, since the pandemic, there have been non-significant differences between women and men in the risk of experiencing aggression. This finding is consistent with some publications reporting that women and men had a similar risk of aggression during the pandemic (18) but contradicts other studies reporting that both women (25) and men (26) were frequently more affected by WPA. Indeed, there is a lack of a clear understanding regarding gender-based differences in the risk of experiencing WPA. This is largely due to the topic not being analyzed in depth, as demonstrated by two recent reviews on the subject, in which only one-third of the included studies examined gender differences (8, 17). Thus, our study provides results on this rather unexplored topic, which needs further research. Our findings on the occupational characteristics of HCW throughout the study period revealed that workers younger than 30 years had a significantly higher rate of WPA, likely because they have a shorter length of service and less training; this finding is consistent with other publications mentioning older age as a protective factor against the likelihood of experiencing WPA during the pandemic (18, 23). In addition, the incidence among nurses and aides was significantly higher than that among other professional categories (among female nurses and aides, the incidence was higher but not statistically significant), which is consistent with the scientific literature (27). It is worth noting that workers in psychiatric and drug dependence centers had the highest incidence, as reported in a recent review (8). Finally, we found that verbal WPA was the most frequent form of aggression, both before and during the pandemic, and these results are consistent with the literature (16).

### Limitations and strengths

This study has the following limitations. First, we did not include repeat incidents of WPA in a single professional. This decision involved a loss of cases but was justified to avoid increasing the incidence artificially because we considered that cases of repeat aggression should be analyzed separately. Second, we considered the entire year of 2020 as the “COVID-19 baseline,” even though the pandemic started at the end of February. Furthermore, we were unable to analyze some key aspects of aggression such as its triggers. Finally, there is a potential source of self-reported bias because all reports of WPA were made by HCWs, which may have led us to underestimate the incidence of WPA.

The main strengths of this study are the large sample that was followed up for 3 years, allowing us to see the impact of the pandemic on the incidents of registered aggression. Furthermore, we stratified the analyses by gender since there is a gender difference in the characteristics of WPA and how these episodes are described. The data sources for this study were reliable administrative and health data that were already collected, providing relevant information on the rarely analyzed impact of the pandemic.

## Conclusions

The COVID-19 pandemic led to an unexpected decrease in the incidence of WPA against PSMar HCWs. Nevertheless, only 5% of these workers experienced at least one unacceptable incidence of WPA in the last year of follow-up. If we considered that WPA is highly underreported in the healthcare environment (10) and that experiencing aggression is associated with both clinically relevant depressive symptoms and psychological distress among HCWs (22), this result is even more significant. In addition, women, young workers, nurses, and workers in the psychiatric center were identified as vulnerable groups with a higher incidence of WPA. Therefore, hospital administrators need to focus on three different factors that mutually reinforce each other to reduce the incidence of workplace aggression. First, they should improve their surveillance and reporting systems to more effectively capture data on incidents of aggression. Second, they should develop robust prevention strategies at the community level, such as education and training programs, to reduce or eliminate aggressive behavior at work. They can also provide training to HCWs on the proper management and identification of WPA, counseling, and psychological help. Third, administrators can implement intervention strategies to prevent both physical and verbal aggression [which are the most frequent forms and have been associated with an invisible psychological effect (28)]. By establishing preventive measures, healthcare workers may be more likely to report incidents of WPA, as they will perceive that it is valued and supported.

## Data availability statement

The raw data supporting the conclusions of this article will be made available by the authors, without undue reservation.

## Ethics statement

The studies involving human participants were reviewed and approved by PSMar Ethics Committee (Project ID 2022/10465). Written informed consent for participation was not required for this study in accordance with the national legislation and the institutional requirements.

## Author contributions

AD: conceptualization, methodology, formal analysis, and writing. MU: conceptualization, methodology, software implementation, formal analysis, writing, and visualization. PD and JM: data collection and writing-review. JR: writing-review. CS: conceptualization and writing-review. FB: conceptualization, writing-review, and supervision. All authors contributed to the article and approved the submitted version.

## References

[1] ILO/ICN/WHO/PSI. Framework Guidelines for Addressing Workplace Violence in the Health Sector. Geneva: ILO/ICN/WHO/PSI (2002).

[2] Krug EG Mercy JA Dahlberg LL Zwi AB. The world report on violence and health. Lancet. (2002) 360:1083–8. 10.1016/S0140-6736(02)11133-0 12384003

[3] ILO. Workplace Violence in Services Sectors and Measures to Combat this Phenomenon. Geneva: ILO (2002). Available online at: https://www.ilo.org/global/topics/safety-and-health-at-work/normative-instruments/code-of-practice/WCMS_107705/lang–en/index.htm (accessed July, 2022).

[4] NIOSH. Violence: Occupational Hazards in Hospitals. Cincinnati: NIOSH (2020). Available online at: https://www.cdc.gov/niosh/docs/2002-101/ (accessed July, 2022).

[5] Civilotti C Berlanda S Iozzino L. Hospital-based healthcare workers victims of workplace violence in Italy: a scoping review. Int J Environ Res Public Health. (2021) 18:5860. 10.3390/ijerph18115860 34072551 PMC8198045

[6] Gascon S Leiter MP Andrés E Santed MA Pereira JP Cunha MJ. The role of aggressions suffered by healthcare workers as predictors of burnout. J Clin Nurs. (2013) 22:3120–9. 10.1111/j.1365-2702.2012.04255.x 22978353

[7] Vidal-Martí C Testor CP. Is Chappell and Di Martino's interactive model of workplace violence valid? An article analysing workplace violence towards healthcare professionals in Spain. Aggress Violent Behav. (2017) 35:83–90. 10.1016/j.avb.2017.05.006

[8] Liu J Gan Y Jiang H Li L Dwyer R Lu K. Prevalence of workplace violence against healthcare workers: a systematic review and meta-analysis. Occup Environ Med. (2019) 76:927–37. 10.1136/oemed-2019-105849 31611310

[9] Raveel A Schoenmakers B. Interventions to prevent aggression against doctors: a systematic review. BMJ Open. (2019) 9:e028465. 10.1136/bmjopen-2018-028465 31530592 PMC6756459

[10] Arnetz JE Hamblin L Ager J Luborsky M Upfal MJ Russell J. Underreporting of workplace violence: comparison of self-report and actual documentation of hospital incidents. Workplace Health Saf. (2015) 63:200–10. 10.1177/2165079915574684 26002854 PMC5006066

[11] Nowrouzi-Kia B Chai E Usuba K Nowrouzi-Kia B Casole J. Prevalence of type II and type III workplace violence against physicians: a systematic review and meta-analysis. Int J Occup Environ Med. (2019) 10:99. 10.15171/ijoem.2019.1573 31325293 PMC6708400

[12] Gerada C. Clare Gerada: some good must come out of COVID-19. BMJ. (2020) 20:369. 10.1136/bmj.m2043 32457205

[13] Devi S. COVID-19 exacerbates violence against health workers. Lancet. (2020) 396:658. 10.1016/S0140-6736(20)31858-4 32891198 PMC7470723

[14] Sheather J Hartwell A Norcliffe-Brown D. Serious violations of health workers' rights during pandemic. BMJ. (2020) 370:m2824. 10.1136/bmj.m2824 32665310

[15] Bhatti OA Rauf H Aziz N Martins RS Khan JA. Violence against healthcare workers during the COVID-19 pandemic: a review of incidents from a lower-middle-income country. Ann Glob Health. (2021) 87:3203. 10.5334/aogh.3203 33977084 PMC8064297

[16] Dye TD Alcantara L Siddiqi S Barbosu M Sharma S Panko T. Risk of COVID-19-related bullying, harassment and stigma among healthcare workers: an analytical cross-sectional global study. BMJ Open. (2020) 10:e046620. 10.1136/bmjopen-2020-046620 33380488 PMC7780430

[17] Ramzi ZS Fatah PW Dalvandi A. Prevalence of workplace violence against healthcare workers during the COVID-19 pandemic: a systematic review and meta-analysis. Front Psychol. (2022) 13:2348. 10.3389/fpsyg.2022.896156 35712196 PMC9195416

[18] Bitencourt MR Alarcão ACJ Silva LL Dutra ADC Caruzzo NM Roszkowski I. Predictors of violence against health professionals during the COVID-19 pandemic in Brazil: a cross-sectional study. PLoS ONE. (2021) 16:e0253398. 10.1371/journal.pone.0253398 34138953 PMC8211185

[19] Zeger S Liang K. Longitudinal data analysis for discrete and continuous outcomes. Biometrics. (1986) 42:121–30. 3719049

[20] Qi M Hu X Liu J Wen J Hu X Wang Z. The impact of the COVID-19 pandemic on the prevalence and risk factors of workplace violence among healthcare workers in China. Front Public Health. (2022) 10:846. 10.3389/fpubh.2022.938423 35958846 PMC9358256

[21] Ghahramani S Lankarani KB Yousefi M Heydari K Shahabi S Azmand S. systematic review and meta-analysis of burnout among healthcare workers during COVID-19. Front Psychiatry. (2021) 12:758849. 10.3389/fpsyt.2021.758849 34858231 PMC8631719

[22] Moro MF Calamandrei G Poli R di Mattei V Perra A Kurotschka PK. The impact of the COVID-19 pandemic on the mental health of healthcare workers in Italy: analyzing the role of individual and workplace-level factors in the reopening phase after lockdown. Front Psychiatry. (2022) 13:1067. 10.3389/fpsyt.2022.867080 35722544 PMC9200968

[23] Pérez-Fuentes MDC Molero Jurado MDM Martos Martínez Á Simón Márquez MDM Oropesa Ruiz NF Gázquez Linares JJ. Cross-sectional study of aggression against Spanish nursing personnel and effects on somatisation of physical symptoms. BMJ Open. (2020) 10:34143. 10.1136/bmjopen-2019-034143 32152167 PMC7064063

[24] Magnavita N. Workplace violence and occupational stress in healthcare workers: a chicken-and-egg situation—results of a 6-year follow-up study. J Nurs Schol. (2014) 46:366–76. 10.1111/jnu.12088 24754800

[25] Zampieron A Galeazzo M Turra S Buja A. Perceived aggression towards nurses: study in two Italian health institutions. J Clin Nurs. (2010) 19:2329–41. 10.1111/j.1365-2702.2009.03118.x 20550621

[26] Camerino D Estryn-Behar M Conway PM van der Heijden BIJM Hasselhorn HM. Work-related factors and violence among nursing staff in the European NEXT study: a longitudinal cohort study. Int J Nurs Stud. (2008) 45:35–50. 10.1016/j.ijnurstu.2007.01.013 17362960

[27] Mento C Silvestri MC Bruno A Muscatello MRA Cedro C Pandolfo G. Workplace violence against healthcare professionals: a systematic review. Aggress Violent Behav. (2020) 51:303–12. 10.1016/j.avb.2020.101381

[28] Beattie J Griffiths D Innes K Morphet J. Workplace violence perpetrated by clients of health care: a need for safety and trauma-informed care. J Clin Nurs. (2019) 28:116–24. 10.1111/jocn.14683 30300949

