# Supplementary material for: Workplace aggression against healthcare workers in a Spanish healthcare institution between 2019 and 2021: The impact of the COVID-19 pandemic
Source: Front Public Health. 2023 Mar 22;11:1070171. doi: 10.3389/fpubh.2023.1070171 (PMC10073706; doi:10.3389/fpubh.2023.1070171)
Supplement: Supplementary file 1 [file Data_Sheet_1.PDF]

Supplementary Figure 1. Flowchart of cohort construction.

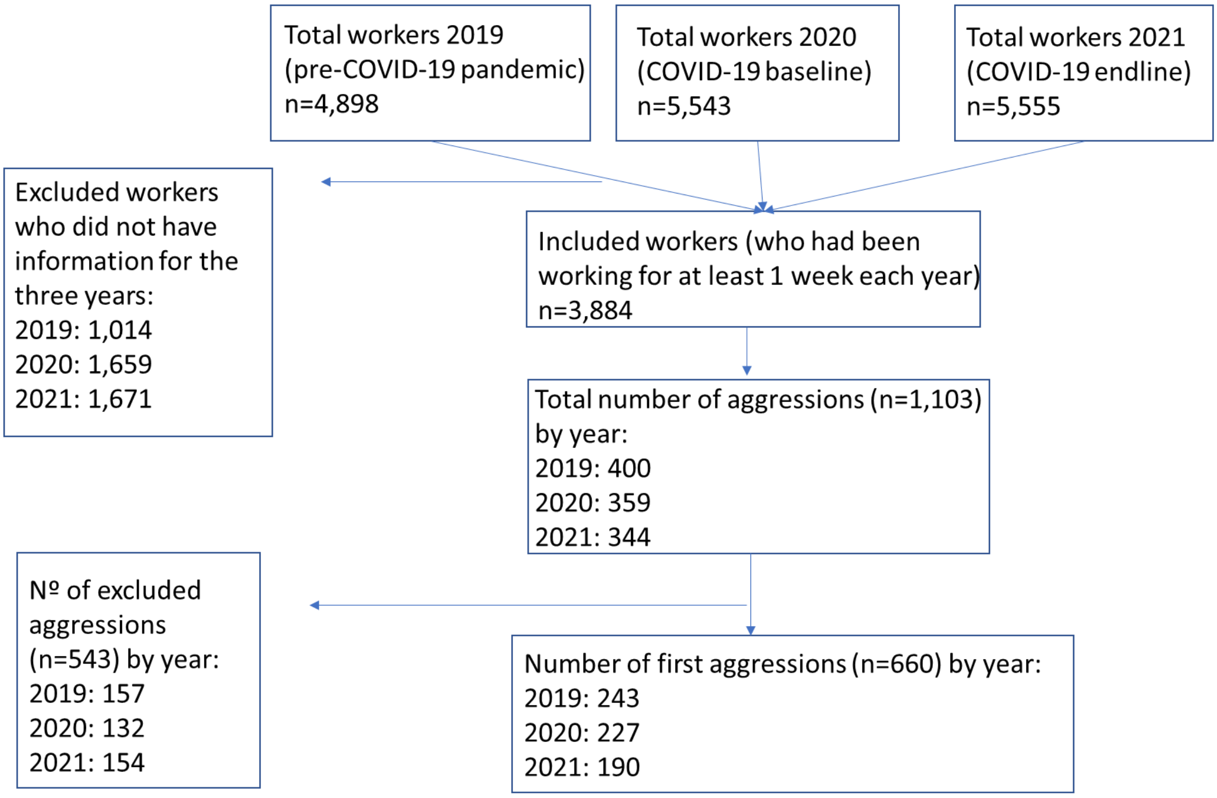

Table s1. Number of HCWs included in the initial cohort and number of HCWs excluded for the analysis, by time period, occupational category and sex.

		Women			Men		
		**Pre-COVID-19 (2019)**	**COVID-19 baseline (2020)**	**COVID-19 endline (2021)**	**Pre-COVID-19 (2019)**	**COVID-19 baseline (2020)**	**COVID-19 endline (2021)**
**Total HCW**	**Physicians**	458	470	489	352	367	362
	**Nurses and aides**	2,238	2,407	2,423	377	427	421
	**Other healthcare workers**	463	628	554	332	376	385
	**Administration and management staff**	469	561	601	209	307	320
**Excluded HCW**	**Physicians**	92	92	88	75	86	71
	**Nurses and aides**	454	620	635	90	142	132
	**Other healthcare workers**	120	299	249	68	113	135
	**Administration and management staff**	83	176	216	32	131	145

Table s2. Incident episodes (n, %), cumulative Incidence (I) and 95% Confidence Intervals (95%CI) of type of aggressions, by time period* and sex.

		Women						Men					
		Pre-COVID-19		COVID-19 baseline		COVID-19 endline		Pre-pandemic		Pre-COVID-19		COVID-19 baseline	
		n (%)	I (95%CI)	n (%)	I (95%CI)	n (%)	I (95%CI)	n (%)	I (95%CI)	n (%)	I (95%CI)	n (%)	I (95%CI)
**Total**		197	6.8 (6.0; 7.8)	174	6.0 (5.2; 7.0)	146	5.1 (4.3; 5.9)	46	4.6 (3.4; 6.1)	53	5.3 (4.1; 6.8)	44	4.4 (3.3; 5.8)
**Type of aggression**	**Verbal**	136 (69.0)	4.7 (3.9; 5.5)	113 (64.9)	3.9 (3.2; 4.7)	95 (65.1)	3.3 (2.6; 4.0)	25 (54.3)	2.5 (1.5; 3.5)	30 (56.6)	3.0 (1.9; 4.1)	31 (70.5)	3.1 (2.0; 4.2)
	**Physical**	61 (31.0)	2.1 (1.6; 2.7)	61 (35.1)	2.1 (1.6; 2.7)	51 (34.9)	1.8 (1.3; 2.3)	21 (45.7)	2.1 (1.2; 3.0)	23 (43.4)	2.3 (1.3; 3.3)	13 (29.5)	1.3 (0.5; 2.0)

*Pre-COVID-19, COVID-19 baseline and COVID-19 endline
